# Addressing the Syndemic Relationship between Lymphatic Filariasis and Mental Distress in Malawi: The Potential of Enhanced Self-Care

**DOI:** 10.3390/tropicalmed9080172

**Published:** 2024-07-31

**Authors:** Carrie Barrett, John Chiphwanya, Dorothy E. Matipula, Janet Douglass, Louise A. Kelly-Hope, Laura Dean

**Affiliations:** 1 Centre for Neglected Tropical Disease, Department of Tropical Disease Biology, Liverpool School of Tropical Medicine, Pembroke Place, Liverpool L3 5QA, UK; 2 National Lymphatic Filariasis Elimination Programme, Ministry of Health, P.O. Box 30377, Lilongwe 3, Malawi; 3 College of Public Health, Medical and Veterinary Sciences, James Cook University, Townsville, 1 James Cook Drive, Douglas, QLD 4811, Australia; 4 Department of Livestock and One Health, Institute of Infection, Veterinary and Ecological Sciences, University of Liverpool, 146 Brownlow Hill, Liverpool L3 5RF, UK

**Keywords:** lymphatic filariasis, mental health, mental distress, self-care, lymphoedema management, disease management, disability, inclusion, gender, poverty

## Abstract

Lymphatic filariasis (LF) causes disfiguring and disabling lymphoedema, which can lead to mental distress and requires life-long self-care treatment. This study applies syndemic theory to understand the biosocial relationship between LF and mental distress in Malawi. Using in-depth qualitative methods, we critically evaluate experiences of mental distress and LF through 21 life-history interviews, to narrate experiences from the perspective of persons affected by LF, and to understand how enhanced self-care (ESC) for lymphoedema management disrupts the syndemic relationship. Complementary key informant interviews with Ministry of Health LF programme staff were conducted to further understand intervention and health system delivery. All interviews were recorded, transcribed, and translated, and then subject to thematic analysis. Our findings suggest that for persons affected by LF in Malawi, before being trained in ESC, absent referral pathways, inequalities in healthcare provision or available treatment, and limited knowledge of the condition (LF) drove the syndemic of LF and mental distress. Distress was often exacerbated by stigma and social exclusion, and shaped by intersections of gender, generation, poverty, and extreme climate conditions. We argue that addressing the syndemic suffering associated with LF and mental distress through interventions which center the needs of persons affected is critical in effective and equitable LF care delivery.

## 1. Introduction

### 1.1. What Are Syndemics?

Syndemics can be described as the clustering of two (or more) diseases or health conditions in a population, which often emerge in conditions where health, social, and economic inequalities are persistent. Such inequalities increase physical and behavioural vulnerabilities within populations, especially among low-income and marginalised groups, often resulting in adverse disease interactions which can be social, behavioural, or biological [1,2]. Stigma, as a social factor, can drive synergistic interactions between diseases, which further complicate the recognition, treatment, and prevention of disease [3]. In this paper, we consider the clustering of health conditions as a result of social and economic disadvantage, an aspect that is often overlooked within biological and social epidemiology. The phenomenon of disease clustering is well evidenced, as is the role of social determinants in explaining differences seen in disease within and between populations [4,5]. However, understanding how the biosocial dynamics of disease can cause or exacerbate disease–disease interaction is lacking [2,6].

### 1.2. Lymphatic Filariasis, Acute Attacks, Disability, and Mental Distress

Lymphatic filariasis (LF) is a neglected tropical disease (NTD) caused by a filarial parasite, transmitted via mosquitoes [7]. An estimated 882 million people in 44 countries remain threatened by LF infection, with 36 million individuals living with LF clinical symptoms. LF leads to disfigurement and disability, which predominantly affect low-income and marginalised populations [7,8]. The second most common clinical symptom of LF is lymphoedema (swelling of the limb or limbs), after hydrocoele (scrotal swelling). Persons affected by lymphoedema are prone to experiencing acute episodes of adenolymphangitis (ADLs), also known as acute attacks, which consist of fever, headaches, and localised inflammation from secondary infection of the already damaged lymphatics [9,10]. Acute attacks have been described as a major contributor to disability and LF severity, as well as causing pain, hindering mobility, and negatively impacting the ability to practice self-care and work, contributing to a loss of earnings [11,12]. Intersections between LF, disability, and mental ill-health are becoming increasingly recognised and evidenced globally [13,14,15,16]. Specifically, disability and acute attacks have been found to be associated with higher levels of depression amongst people affected by LF and other skin NTDs [17,18]. However, generally when considering the interaction of mental health conditions and LF, the focus is on epidemiological clustering, with the biosocial context less considered [19,20,21,22,23,24]. This is despite widespread recognition that understanding the biosocial connections between diseases is crucial in responding to disease interactions [1,13,25,26]. In Malawi, there is evidence highlighting the co-morbidity of LF and mental ill-health based on epidemiological clustering [18]. Current estimates suggest just under 30,000 people are living with symptoms of LF in Malawi that require holistic (physical, mental, and social) care [27].

### 1.3. WHO 2030 Targets and Disease Management, Disability, and Inclusion (DMDI) Approaches

As outlined in the World Health Organization (WHO) NTD roadmap 2021–2030, a shift from vertical programme delivery to an integrated holistic approach is required to address the physical, mental, and social consequences of disease and maximise resources. The roadmap also highlights the importance of considering the needs of persons affected within elimination and disease control interventions [28]. As Malawi and other LF-endemic countries progress toward the elimination of transmission of LF through the successful delivery of preventative chemotherapy, the focus must shift toward delivering morbidity management and disability prevention (MMDP) services for people with existing disease [29,30]. Currently, the scope of most national MMDP programmes is limited to the provision of hydrocoele surgeries and training for lymphoedema management [31]. Within recent years, a more holistic concept of MMDP has been promoted amongst NTD practitioners, described as disease management, disability, and inclusion (DMDI) [32]. This has an emphasis that’ disability’ is caused by a condition or impairment within a particular context, and that social manifestations, and other often non-medicalised consequences such as mental health and stigma, are considered as contributors to disability. Additionally, ‘inclusion’ reflects the need to include persons affected within programme design and implementation. Thus, considering the psychosocial consequences of LF, perspectives of persons affected and the wider biosocial context are essential to achieving person-centred holistic MMDP, henceforth described as DMDI [33].

### 1.4. The Context of Malawi and the National LF Programme

In Malawi, LF is widespread, with 26/28 endemic districts. The economy is largely reliant on agriculture (over 80%), causing vulnerabilities to extreme climate conditions that may result in damage in agricultural production, e.g., poor crop harvest [34]. The Malawi National LF Elimination Programme successfully achieved the validation of the elimination of LF as a public health problem from the WHO in 2020 [29]. Elimination was largely achieved through the completion of multiple rounds of preventative chemotherapy through mass drug administration (MDA) in all endemic regions [35]. A key challenge facing the programme moving forward is to deliver the major component of the Global Programme to Eliminate LF (GPELF) strategy, which requires the implementation of MMDP for all those living with the clinical symptoms of LF. Within seven districts in Malawi, a home-based ‘enhanced self-care’ (ESC) for lymphoedema management has been integrated within the health system through the training of primary health staff [33].

### 1.5. Enhanced Self-Care Study

In three highly endemic districts for LF in Malawi where the ESC was integrated into the health system, a 6-month prospective study was conducted in 2021, implementing an ESC intervention for lymphoedema management [36]. The study involved training persons affected with LF-related lymphoedema and their primary caregiver in ESC. This included standard WHO-recommended activities including hygiene and skin care practices, daily and overnight elevation of affected limb(s), seated and standing exercises, managing acute attacks with medication, and wearing of appropriate footwear [37]. Enhanced self-care activities included deep-breathing techniques, lymphatic massage, skin mobilisation, walking, drinking water, and eating fresh fruit and vegetables [36]. Activities were designed to impose no financial burden on participants and were easy to practice independently in order to improve physical symptoms of lymphoedema, reduce acute attacks, and improve quality of life.

### 1.6. Study Contribution and Rationale

This paper presents the use of in-depth qualitative methodology to explore the syndemic relationship between LF and mental distress in Malawi. Drawing on Mendenhall’s model of syndemic approaches to health [2], through narratives of persons affected by LF, we consider the following: (1) the lived experiences of the syndemic and current healthcare response; and (2) the impact of an enhanced self-care intervention on the syndemic relationship, with a view to make recommendations to improve the mental and physical wellbeing of people affected by LF in Malawi. This is the first study to consider the syndemic relationship between LF and mental health from the perspective of persons affected in Malawi. Additionally, this research aims to understand how ESC can support health systems to manage LF and mental wellbeing for people affected by LF, focusing specifically on how ESC disrupts this syndemic relationship.

## 2. Materials and Methods

Life history interviews were conducted with 21 persons affected by LF-related lymphoedema or both hydrocoele and lymphoedema, to explore the syndemic relationship between mental distress and LF, and the impact of the ESC intervention from the perspective of persons affected. In this paper, we refer to mental distress as a continuum of descriptions from stress, anxiety, and depression, to suicidal thoughts within the narratives of persons affected. To complement life history narratives and better understand the context of implementing the ESC within the Malawian health system, key informant interviews were conducted with LF programme staff and LF experts in Malawi.

### 2.1. Study Setting and Design

This study was conducted in Chikwawa district, Malawi in 2022. This study followed-up a subset of participants who were part of a wider prospective cohort study and trained in ESC conducted in three sites, Karonga (Northern), Nsanje, and Chikwawa (Southern) districts, in Malawi over a 6-month period in 2021. This wider study investigated the physical impact of the ESC intervention for LF-related lymphoedema. Chikwawa was selected as the study site for this qualitative study due to accessibility and finances available and because of the high prevalence of clinical cases of lymphoedema identified in this district. Participants recruitment period was from 2 August 2022 to 11 August 2022.

### 2.2. Participants, Sampling Procedure, and Data Collection

#### 2.2.1. Life Histories

The use of life history interviews allowed persons affected to narrate their lives and experiences of illness, repositioning them as a storyteller that is best placed to recount their own reality of suffering and resilience. Furthermore, narratives can support individuals to make sense of their illness experience [38,39].

Persons affected by LF-related lymphoedema were purposively selected for life history interviews to ensure maximum variation of characteristics, see sampling matrix in Table 1. Sampling criteria were selected based upon findings from a previous study, identifying associations with higher number of acute attacks and depressive symptoms [18]. This previous study was used to categorise participants into either a “mental distress” group who had recorded a depressive symptoms score (PHQ-9) of above 5, or from descriptions of mental distress collected from an open-ended question: “tell me about your mental health?” where responses were indicative of poor or unchanged mental wellbeing. Depressive symptoms scores were assessed using the Patient Health Questionnaire (PHQ-9) over the study time period of 6 months [18,40]. All other participants fell into the “mental wellbeing improvement” group, indicated from an improvement of depressive symptoms. The change in number of acute attacks was also derived from the same previous study.

Sample size was estimated based on the number of life history interviews that was anticipated to reach saturation based on previous research exploring the biographical accounts of persons affected by NTDs. Through our iterative and ongoing approach to analysis, we determined data saturation was reached within our sample and data collection finalised [41]. All persons affected were recruited for this qualitative study due to their previous enrolment in the previous ESC study [18] and were identified through local health facility registers and national programmatic records. One primary caregiver was not enrolled on the ESC study but had received lymphoedema training as part of the ESC study and was therefore included in the life history interviews. Characteristics described in Table 1 were summarised for each participant within Table S1.

Life history interviews took place at the homes of the participants and interview guides were developed prior to interviews aiming to explore the main themes, life histories, experiences of LF (somatic experience), mental wellbeing, stigma, enablers and barriers to the ESC, health-seeking, participation, additional support (i.e., caregivers), and life after the ESC study. A second Malawian researcher (DEM) conducted life history interviews in Chichewa, and translated in real-time to CB to allow follow-up questions. All data collected were audio recorded, transcribed verbatim, and translated into English. To maintain the originality and clarity of information during transcription and translation, 10% of transcripts were randomly selected and cross-checked against audio by DEM for quality assurance purposes.

#### 2.2.2. Key Informant Interviews

Key informant interviews were purposively selected based upon their previous involvement in implementing the ESC study (unpublished data) [18], which included three Ministry of Health (MoH) staff members. The lead author (CB) conducted all key informant interviews in English, which were audio-recorded and then transcribed verbatim.

CB read through all transcripts to ensure completeness for data analysis.

### 2.3. Data Analysis

**Figure 1:**
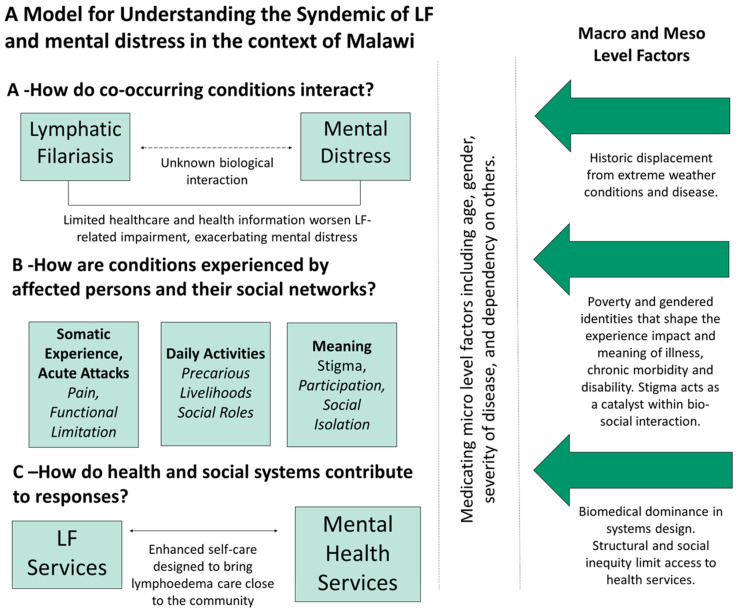
A Model for Understanding the Syndemic of LF and Mental Distress in the Context of Malawi.

All data analysis was guided by framework adapted from Dean et al.’s (2023) describing the syndemic model for NTDs and mental distress, which was informed by Mendenhall’s (2017) model of syndemic approaches to health (see Figure 1) [2,15]. This study adapted the framework developed by Dean et al. (2022) that describes the syndemic relationship between NTDs and mental distress in Liberia. Dean et al.’s (2022) original model was informed by Medenhall’s (2017) model of syndemic approaches to health. As such, the model of LF and mental distress presented here articulates three main dimensions driving this syndemic within Malawi: (A) the epidemiological co-occurrence and interaction of LF and mental distress; (B) the experiences of persons affected by LF and mental distress grouped into somatic, daily activities, and meaningful experiences; and (C) how the ESC, health, and social systems contribute to responses in LF care. We were specifically exploring how the ESC that was designed to bring lymphoedema care close to the community disrupts this syndemic of LF and mental distress. Thematic analysis was conducted using a deductive coding approach based on topic guides and the adapted framework(s) and additional themes emerging from the data were inductively coded. Analysis was supported by NVivo 12 software.

### 2.4. Ethical Statement

Ethical approval was obtained from the Liverpool School of Tropical Medicine, Research Ethics Committee, UK (Research Protocol 22-036) and National Health Sciences Research Committee, Malawi (Number 2615). Written informed consent was obtained from all participants in this study. Consent forms and information sheets, describing the study aims and data collection processes, were translated into Chichewa, the local language, and read out to participants who were less literate. All participants had the opportunity to ask questions and or discuss queries to participation.

## 3. Results

The pathways reinforcing the syndemic relationship between LF and mental distress in Malawi are shown in Figure 1. We present the study findings aligned to the adapted framework (Figure 1), across three main dimensions: (A) ‘How do LF and Mental Distress Interact?’; (B) ‘How are Conditions Experienced?’; and (C) ‘How do Health and Social Systems Contribute to Responses in LF and Mental Health Services?’. This study found that multiple pathways have played a role in the progression of LF symptoms (especially lymphoedema), disability, chronic illness, and mental distress, largely as a result of underlying health and social inequalities.

**Figure 2:**
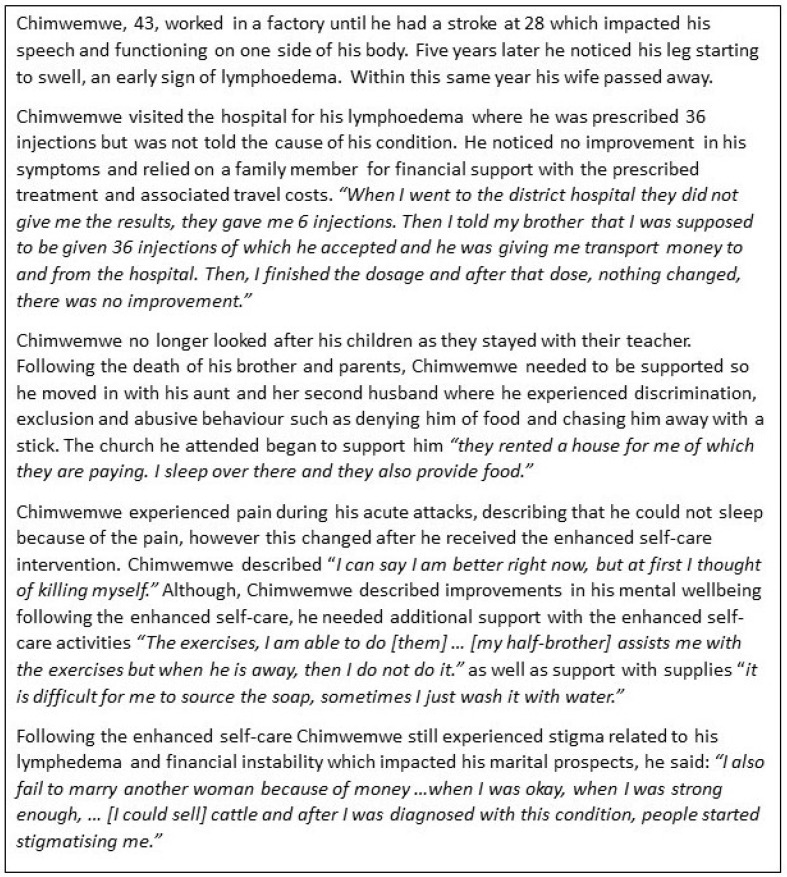
Chimwemwe’s story.

**Figure 3:**
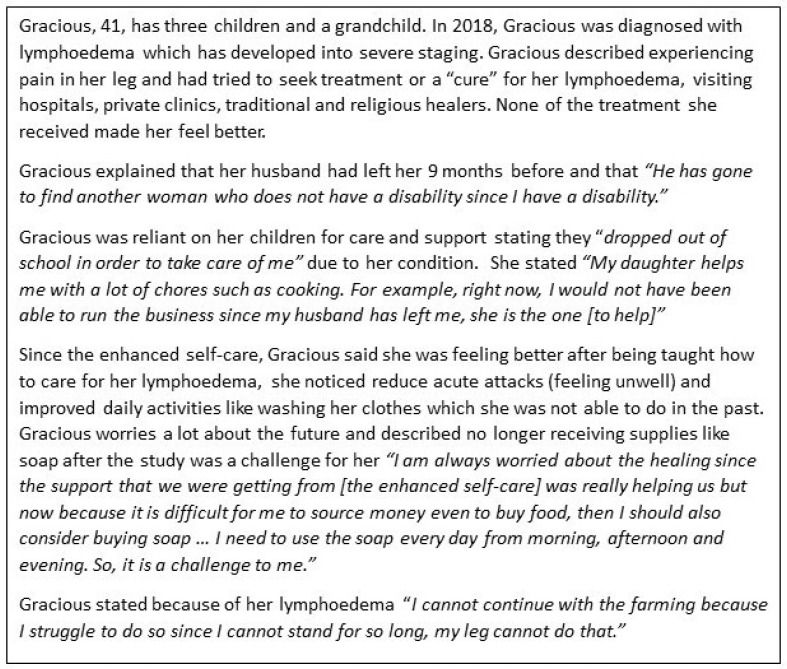
Gracious’ story.

Before exploring the syndemic, we first present two narratives from persons affected. Both Chimwemwe’s (Figure 2) and Gracious’s stories (Figure 3) capture syndemic suffering related to LF and mental distress and highlight how the wider social and environmental context (such as weak health and social systems) intersect with gender, poverty, and disability to exacerbate physical morbidity and mental distress.

### 3.1. (A) How Do Co-Occurring Conditions Interact?

The first main dimension of the syndemic model of LF and mental distress (Figure 1) explores (A) the co-occurrence and interaction of LF and mental distress. Within this section we describe the interaction between LF and mental distress, the drivers of this related to discovery of lymphoedema, and healthcare availability. Finally, the coping strategies, social systems, and religious support that can disrupt this interaction between LF and mental distress are described.

#### 3.1.1. How Do LF and Mental Distress Interact?

Barrett et al. (2023) explored the co-occurrence of LF and mental health conditions in Malawi. Of 311 people affected by LF-related lymphoedema, 20.3% (95% CI 15.9 to 25.2) reported mild depressive symptoms and 3.2% (95% CI 1.5 to 5.8) reported moderate depressive symptoms assessed using the Patient Health Questionnaire (PHQ-9). For QOL, 28.6% (95% CI 23.7 to 34.0) reported moderately low QOL and 2.9% (95% CI 23.7 to 34.0) reported severely low QOL, using the LF-specific Quality of Life Questionnaire (LFSQQ) [18]. Furthermore, rates of mental distress amongst people affected by LF have been shown to be higher than in the general population in other countries [23,42]. Evidence of the biological interaction between LF and mental distress is lacking, although indication of the bidirectional relationship between depression and inflammation [43] and the social and psychological consequences caused by cancer-related lymphoedema [44] have been made. Within this paper we discuss how the social consequences (particularly around stress-causing stigma and the internalisation of stigma) associated with LF, contribute to the syndemic of concern. Amongst other diseases, stigma and stress can contribute and enhance mental distress, specifically depression and anxiety [3,45]. We suggest that the social consequences of LF, such as stigma (internalised and enacted) and stress related to their condition, are responsible for the interaction of LF and mental distress and exacerbated by progression of disease and degree of disability.

#### 3.1.2. Discovering Lymphoedema and Healthcare Availability

We found that limited knowledge of lymphoedema at the point of diagnosis or symptom onset, alongside the chronic, progressive, and disabling nature of lymphoedema caused mental distress. This was exacerbated by the absence of appropriate healthcare and lack of available treatments or a “cure” for their often-unknown condition. Levels of mental distress varied amongst participants, from feelings of worry, hopelessness, and stress to descriptions of common mental health conditions such as excessive worry (anxiety) and, for some, suicidal ideation.

“*When I discovered that I have got this problem, my heart sometimes stresses out … I could think of just dying because I was feeling like I am a failure*”.

***(Female, 49, Participant 5)***

#### 3.1.3. Coping, Social Systems, and Religious Support

Faith played an important role in coping with LF and mental distress, particularly in times of depression or suicidal ideation. Faith-based organisations offered social support for persons affected; for example, Chimwemwe described the religious group he attended provided him with housing after being ‘chased’ out of the house he was staying in by a relative, as well as providing him with psychological support from religious group members.

“*When my husband left me … I just told God, [and] everything came back to normal. And some people also helped me praying over it, so yes, I am okay*”.

***(Female, 40, Participant 20)***

Religious teachings promoted acceptance or “being at peace” with their condition or difficult life circumstances, whilst some described increased shame related to suicide ideation as this was a “sin”.

“*… I thought of killing myself. Then I had to comfort myself that I should [not] do that because the bible tells us that committing suicide is a sin and I just accepted to live and struggle like that*”.

***(Male, 43, Participant 12)***

Outside religious groups, ways of coping with LF, mental distress, and, in some cases, suicidal ideation, were described as self-distraction, community support through talking, and reassurance. Some participants also described alcoholism or a belief ‘that others will develop the same condition one day” as coping processes.

### 3.2. (B) How Are Conditions Experienced?

The second main dimension of the syndemic model of LF and mental distress (Figure 1) explores (B): How are Conditions Experienced? Within this section, we discuss how persons affected by LF experience the multi-directional interactions between somatic experience, the ability to complete daily activities, and the social meaning of disease (stigma, participation, and social isolation).

#### 3.2.1. Somatic Experience and Acute Attacks

Somatic experience varied across life-history narratives, depending on pain, functional limitation, and changes in physical appearance, which caused mental distress. For many, prior to the ESC intervention, functional limitations and alterations to physical appearance were more permanent. However, during the first onset of symptoms, this was more transient or temporary. The greatest levels of pain and functional limitation (somatic experience) were experienced during acute attacks. Some described being completely immobile, unable to move from their bed to urinate. These periods were closely associated with experiences of extreme mental distress and many described suicidal ideations during these episodes.

“*I usually think about … [suicide] because of the pain … I feel it is better to die than just to suffer*”.

***(Female, 36, Participant 9)***

#### 3.2.2. Daily Lives and the Impact of Acute Attacks on Household

Loss of job opportunities, dependency on others, and inability to meet gender roles contributed to mental distress, particularly feelings of failure and worry, due to the somatic experience (physical limitations and pain) associated with LF. Precarious livelihoods centred around day-to-day survival (subsistence farming, foraging, and selling) were a major focus in narratives and a key trigger of distress, particularly where individuals felt unable to achieve essential survival activities.

“*The depression is there since I have mentioned that I need to search for food so if I do not work, I do not have food. So, food can be unavailable for me to eat and sometimes I am depressed since I do not have any other ways for me to find food*”.

***(Male, 53, Participant 21)***

Households were impacted during acute attacks, due to participants’ dependency on caregivers (often a spouse or family member) and the inability to contribute to household chores or work, such as subsistence farming, which are crucial for day-to-day survival.

“*After being diagnosed … I am not able to do most of the chores that I used to do in the past … I am a failure because for … [someone] who does not have this condition … is able to work properly … while for me I have a limitation*”.

***(Male, 51, Participant 2)***

“*…when I got better [following acute attack], my friends were already at another level with the farming and that meant that I will not have enough food that year. So, because of that, I could think of just dying because I was feeling like I am a failure*”.

***(Female, 49, Participant 5)***

#### 3.2.3. Social Roles

Here we discuss how social roles, such as the ability to meet gender roles, relationships, and marital prospects, are impacted by LF, which leads to mental distress. Both men and women described their inability to meet expectations of gendered social roles (e.g., as a wife, mother, husband, or father) because of their disability and physical limitation, which led to low self-esteem, feelings of failure, worry, and, in some cases, depression. *“The worrisome moments for me as a man since at times there is no food at home.”* (Male, 39, Participant 19). The importance placed on meeting gender roles was greatest amongst younger participants, as described by one participant: *“If the condition develop[s] while you are old, you cannot be that worried.”* (Female, 39, Participant 8).

Some women described distress related to their ability to raise and provide for offspring. For example, in Gracious’s story, her children acted as the primary caregiver, leading to missed school days or complete suspension of education. For men, this was articulated as a failure to financially provide for their families. Caring responsibilities impacted participation, education, and employment opportunities, highlighting additional gender inequalities associated with lymphoedema where women were largely identified as the primary caregiver [46,47,48]. For women, and some men, the inability to fulfil gendered ideals often reduced marital prospects. However, one participant experienced stigma from her community when courting her now husband, which didn’t impact her marital prospects.

“*If I am to find a partner, she will mock me saying you have a disability … Women stigmatise us because of this and they say the way you are looking with your legs; can we be married and move together? So, it is difficult for me to ask a woman out*”.

***(Male, 53, Participant 21)***

“*Even when my husband was marrying me, people were telling my husband that if you are getting that girl with big legs, you will have troubles with her but he was telling them that only God knows everything. He was interested in me and not in my legs. He wanted marriage and not the legs*”.

***(Female, 65, Participant 14)***

Experiences of verbal or physical abuse behaviours from a spouse due to having lymphoedema were generally more normalised amongst females. However, some men decided to separate from their spouse because of intra-marital stigma enacted through verbal or physical abuse; and others reported additional challenges related to hydrocoele symptoms impacting marital and sex life, though improvements were described following surgery.

“*Ah … [the condition did not affect my marriage] with the current wife but the first wife whom we separated, we were having quarrels … she would tell me … do you think that after leaving me you will get another wife with your condition? … I decide to just leave her*”.

***(Male, 39, Participant 19)***

#### 3.2.4. Meaning of Disease

Within this section, we discuss how the social meaning of disease (stigma, participation, and social isolation) can shape individual experiences. Internal stigma (‘felt’ stigma resulting from either the anticipation of external stigma or internalised stigma/feelings about oneself) or external stigma (actual experiences of stigma described as ‘enacted’ stigma) [49] were identified amongst people affected by LF and often exacerbated mental distress. Experiences of stigma were shaped by and contributed toward individual physical appearance, inability to meet gender roles, functional limitations, inability to work, and financial instability.

##### External (Enacted) Stigma

We found that experiences of verbal insults or segregation from the community were common amongst persons affected and a key driver for mental distress.

“*friends that I used to chat with … had … thoughts that I should not … [go] near them … they could chase me away [and] say get out, you have got lymphoedema. [That] caused me stress … people … [would] say, hey you! Do not chat with this person, he is supposed to be left alone*”.

***(Male, 39, Participant 19)***

Experiences of stigmatising behaviours, including being laughed at, being singled out, being talked about or being avoided, were often not recognised as ‘stigmatising behaviours’. However, some did label extreme levels of discrimination and segregation they experienced from their community as stigmatising. This highlights the differences in definitions of stigma between participants and researchers, or potentially the normalisation of disability-related stigma within participant communities.

Experiences of enacted stigma for those with mild-stage lymphoedema that were less visibly disabled and amongst elderly participants were far less reported. Here, stigma often subsided over time when not particularly problematic at the outset or was only experienced when meeting new community members. Elderly participants reported the fewest experiences of enacted stigma.

“*[People] were pointing fingers at me to say[ing] … she has lymphoedema. So, as a woman, I started feeling shy … of course, there are some people who are just new in the community and they wonder why I have these big legs*”.

***(Female, 39, Participant 8)***

“*No, I was living with them freely [following LF diagnosis] and there was no problem. I was able to chat with them as well. I had my peace of mind even up to now*”.

***(Female, 60, Participant 14)***

Females, those living in poverty, and more visibly disabled individuals recalled the greatest experiences of enacted stigma from the community and in some cases family/relatives. Accounts of challenging enacted stigma were reported by men and not women, potentially attributed to the existing gendered hierarchy. Additionally, such intersections of stigma, gender, poverty, and disability increasingly contributed to mental distress.

“*They were laughing at me … They were telling me that it was the beginning of elephantiasis, and I will not get better, that was the beginning of a disability … [and] the leg will become bigger*”.

***(Male, 47, Participant 4)***

“*When I was okay, when I was strong enough, [I] was selling cattle and after I was diagnosed with this condition, people started stigmatising me*”.

***(Male, 43, Participant 12)***

Some accounts linked stigma with delayed or poor quality of care, as one female described she “was not assisted well” within a healthcare setting. Limited knowledge around causes of lymphoedema contributed to both internal and enacted stigma exacerbating mental distress. Traditional belief systems often exacerbated misinformation around the cause of lymphoedema and associated stigma, rooted within community and personal beliefs which were often reinforced by traditional healers.

“*Some use the supernatural powers when they want to farm … people advised me to stop farming on that field … my penis [started] to swell such that I was struggling with life… [I was advised] to stop using the fields or … [it] might kill me … Later on … my legs started swelling … it started as if I was bewitched only that the legs were not shrinking even if I went to the hospital*”.

***(Male, 47, Participant 4)***

##### Internalised Stigma and Participation

Key drivers of internalised stigma were associated with limited functioning, inability to work, and inability to meet gender social roles (as described above). Greater internalised stigma was associated with repeated experiences of enacted stigma, such as in relationships (abuse or separation) and from community members (social exclusion, verbal insults, or segregation)

“*… my marriage ended because I am sick…to say the way this person is sick, how useful can he be?*”

***(Male, 53, Participant 21)***

Participation, internalised stigma, and mental wellbeing were found to interlink; for example, a lack of participation caused anxiety or worry, described as “stay here and wait” or “thinking a lot”. Participants were negatively impacted by their degree of disability, functional limitation, and physical appearance and experienced enacted stigma. Women seemed to experience more shame than men, although females gave fewer accounts of social exclusion, regardless of their lymphoedema. Some adjusted their behaviours, such as by wearing trousers to cover their lymphoedema “So, as a woman, I started feeling shy and I was wearing a wrapper to cover the leg” (Female, 39, Participant 8).

“*I feel shy sometimes … I am a chief in this village and I have a name but they nicknamed me chief with a big foot, such that when you are to move around you will hear that name amongst people from this area*”.

***(Male, 51, Participant 2)***

### 3.3. Displacement, Poverty, Disease, and Health Inequities

Within our syndemic model of LF and mental distress, we highlight how intersections and influences at the macro and meso level can impact disease interactions and individual experiences. In Malawi, macro and meso factors identified by persons affected were grouped into displacement, poverty, disease, and health inequities. Distressing life events within narratives were described in relation to displacement from annual extreme weather conditions in the Chikwawa. For some, this caused frequent displacement and, when coupled with precarious livelihoods and lymphoedema, caused additional mental distress. As Chimwemwe described, “I was … [planning] to build a house … with this condition, I failed to do that..”. Financial instability and dependency on others made family illness and death additionally distressing, and often visits to formal health settings were associated with stress.

“*that missing person is my brother … he was the one who was taking care of me. … we found him … [dead] … so, from that day, I failed to sleep because … now, I am the only … [family member] remaining*”.

***(Female, 38, Participant 1)***

Additional physical impairments, as well as lymphoedema, resulted in increased dependency on others. This also resulted in extremely poor living conditions and increased vulnerabilities to additional health and social risks.

“*I do [think about dying] because living with this condition, even … [my] guardian gets tired of taking care of me and sometimes if she gets tired, she forgets to provide some other things*”.

***(Female, 62, Participant 16)***

### 3.4. (C) How Do Health and Social Systems Contribute to Responses in LF and Mental Health Services?

In the following section, we discuss the third main dimension of the syndemic model of LF and mental distress, (Figure 1) (C) How do Health and Social Systems Contribute to Responses in LF and Mental Health Services? Firstly, challenges around accessing available LF and mental health services in Malawi prior to the ESC are discussed. Secondly, how the ESC addresses gaps in LF services, and how the ESC disrupts the syndemic of LF and mental distress, specifically drawing on each person’s affected experiences of conditions (somatic, daily activities, and meaning of disease) is discussed. Finally, the sustainability of the ESC is discussed from the perspective of persons affected by LF.

#### 3.4.1. Health Stress, Social, and Structural Barriers

Repeated care-seeking via formal and informal health providers was often a result of weak formal health system capacity to diagnose and treat lymphoedema. This was due to the lack of trained personnel or knowledge of available treatment, unavailable or absent healthcare staff within health facilities, limited supplies, such as painkillers to alleviate acute attacks, and inappropriate and costly treatment which did not improve symptoms. The choice of health-seeking via informal and formal providers was shaped by community or personal beliefs of disease cause and previous care received from providers. Repeated healthcare-seeking visits were often cited as a cause of stress.

“*I have been coming [to the hospital] … several times and the doctor said I can read it from your form*”.

***(Female, 65, Participant 14)***

“*After I came back from the hospital and when I noticed that I was not feeling better, I started visiting the traditional healers*”.

***(Female, 40, Participant 20)***

Health-seeking through the formal health system was common, except for one participant who had only accessed traditional care and a pharmacy. Additionally, some elderly participants did not seek any formal or informal care, highlighting potential generational differences in Malawian health-seeking behaviours.

“*I just accepted that my leg was swollen because all of my parents also died with this same condition … they were not seeking medical care from the hospital as we are doing it right now*”.

***(Female, 65, Participant 14)***

All persons affected that were categorised as “mentally distressed” within the sampling matrix (Table 1) reported repeated care-seeking in the first year of symptom onset in an attempt to improve their condition, exhausting all formal and informal options.

“*I still visit the hospital [as well as traditional healers] and at times I buy medicine from private clinics … I have tried going to pastors for prayers and for blessed water … When I have money, I go and buy the medication and [my leg] … usually shrinks*”.

***(Female, 40, Participant 20)***

#### 3.4.2. Financial Burden of Healthcare-Seeking, Lymphoedema Management, and Misdiagnosis

Repeated care-seeking brought additional financial stress for individuals, households, and extended families, related to travel and treatment costs and days off work to attend health services. This was exacerbated by a dependency on others for travel to health services and financial support. These barriers and financial stressors were greatest for individuals living in economic instability (poverty), particularly if relatives were in similar financial positions. Consequently, these factors led to delaying seeking healthcare and reducing the ability to access health services.

“*When I went to the district hospital … I told my brother that I was supposed to be given 36 injections of which he accepted, and he was giving me transport money to and from the hospital*”.

***(Male, 43, Participant 12)***

Misdiagnosis and inappropriate prescribed treatment resulted from a lack of trained personnel and knowledge around lymphoedema within formal health settings. However, many were prescribed drugs (painkillers or antibiotics) to manage their acute attacks. Numerous injections were prescribed to some younger participants to treat their lymphoedema, which MoH staff stated were often prescribed to treat cancer-related diagnoses. Chimwemwe and others shared their experiences within a formal healthcare setting where they described a likely misdiagnosis and inappropriate treatment, which imposed a potentially unnecessary financial burden upon a person affected and their family (see Figure 1).

“*As a young man … I noticed that my leg has started swelling … I went to the district hospital, … [and] I was prescribed 18 injections …. [I was] failing to withstand the budget of staying at the hospital, it was difficult. I needed food and other things … I continued getting the injection[s] but because I did not have money, I failed to finish all the prescribed injections*”.

***(Male, 39, Participant 19)***

#### 3.4.3. Resource and Capacity Limitations

Amongst those affected by hydrocoele, cancelled hydrocoelectomy procedures were commonly reported due to prioritisation of emergency surgeries, limited surgical supplies, and unavailability of health staff, resulting in increased morbidity, travel expenses, and missed days off work.

“*I was feeling tingling, and pain and I went to the hospital because I was failing to sit on the bicycle since my private part was also swollen … when I went [to the hospital] I was told that I need to be operated. So, they gave me a date and when I went back on that particular date, they told me to go there on another date. I went again, and when I arrived there, after they examined me, they told me that I need to try taking some medication before the operation [and could not have surgery that day]*”.

***(Male, 70, Participant 6)***

### 3.5. Mental Health Service Provision in Malawi

Like most LMICs, current resources allocated for mental healthcare in Malawi are limited, reducing treatment and management options [50,51].

“*There’s no … training for mental health for community health workers and even for the patients I think we overlook this this side of mental health in Malawi … so for the patients with elephantiasis I think we have been neglected in terms of psychosocial support or mental health because we don’t train them or we don’t even help them in any way mentally … … but most of the time when they come with lymphoedema we only look at the clinical part and clinical management*”.

***(Programme Staff Member)***

### 3.6. ESC Addressing Gaps in LF Service Provision

The ESC intervention addressed gaps in LF service provision in Malawi. Douglass et al. (2019) designed the ESC intervention with a biological focus to improve the somatic experience of individuals living with lymphoedema, to impose no added financial burden on persons affected, and to be easy to remember for persons affected and their primary caregivers. Participation in the ESC equipped most with knowledge of their condition and diagnosis, alongside skills to manage their symptoms, particularly during acute attacks. This reduced repeated healthcare-seeking, reducing costs associated with travel and inappropriate treatment.

“*I started practising [the self-care] … things have improved. Right now, I am able to run which was difficult for me in the past, the skin peeling stopped … when I started massaging it … and the exercises … the pain is gone … I was unable even to ride a bicycle … so when [my family] saw me moving about, they were happy*”.

***(Male 47, Participant 4)***

The management of the physical symptoms of LF was anticipated to indirectly improve daily activities and meaning (i.e., stigma and social isolation) of the persons affected. Supplementary Table S1 summarises each domain (activity) of the ESC and the intended impact of the ESC intervention on the syndemic pathways identified. Evidence of ESC outcomes from the participants’ perspective and additional recommendations are also presented.

### 3.7. The Potential for ESC to Disrupt the Syndemic Relationship between Mental Health and LF

Within this section we discuss how the ESC disrupts the syndemic of LF and mental distress, firstly in relation to the interaction of LF and mental distress and then relating to individual experiences of these conditions (somatic, ability to complete daily activities, and the social meaning of disease). Discovering that treatment, supplies, and financial support for lymphoedema self-management was available through the ESC study led to improved wellbeing, particularly for those who repeatedly sought care. Despite overall improvements in mental wellbeing, for some, the chronic nature of lymphoedema and there being no “cure” still caused distress, particularly for those living with severe staging of lymphoedema, multiple disabilities, and the elderly.

#### 3.7.1. Somatic Experience and Acute Attacks

Improvements in somatic experience, such as less pain, decreased frequency and duration of acute attacks, reduced swelling, and wound healing following the ESC were described, which was attributed to improved mental wellbeing and ESC adherence by participants. All persons affected described a reduction in acute attacks following the ESC, which corroborates Barrett et al.’s (2023) findings that higher depressive symptoms were associated with higher numbers of acute attacks. Reduced acute attacks and participants generally feeling “well” reduced adherence to the ESC, though periods of feeling “unwell” during acute episodes reminded participants to continue to practice ESC.

“*since from that time, that you taught us the home-based care, I have not been sick [acute attacks] … I am able to work as you can see*”.

***(Male, 51, Participant 2)***

#### 3.7.2. Daily Activities

Many described a sense of stability in their life following the ESC intervention and that ESC activities were easy to practice within daily life. Livelihood and social activities such as carrying out work (subsistence farming or selling), household activities (chores), and socialising with peers, were barriers to practising the ESC, which was exacerbated by precarious livelihoods and periods of financial instability. This left some participants feeling overwhelmed when balancing lymphoedema care with livelihood activities.

“*Sometimes because of being so overwhelmed with work, I miss out some days without cleaning it*”.

***(Male, 51, Participant 2)***

Improvements in somatic experience, particularly reduced acute attacks and improved functioning, improved participation in daily activities like walking, completing chores, farming, and practising lymphoedema care. These improvements subconsciously improved ESC adherence (i.e., walking and standing exercises). Increased participation enabled the ability to fulfil gender social roles, reduced dependency on others for financial support, and increased days working, thus improving mental wellbeing. However, precarious livelihoods, living with a life-long chronic condition, and the recurrence of dependency on others still caused distress following the ESC.

“*For me to be able to support my children in terms of education … pay[ing] school fees … buying them school uniforms and other necessities … when I fall sick, I am not able to provide all those things to my children … [due to my condition] my life is not the same again*”.

***(Female, 53, Participant 3)***

#### 3.7.3. Meaning of Disease-Stigma

Internalised stigma was reported to reduce following the ESC. Information shared about causes of lymphoedema at the start of the ESC intervention challenged self-stigmatising beliefs around causes of lymphoedema such as being “bewitched” or “cursed”. However, some mental distress was still ongoing and related to limited knowledge and concerns around lymphoedema.

“*I was thinking that maybe I inherited [lymphoedema] from my parents …. it has been a while since I developed this condition even though I am on treatment but things are not going on okay … you might be thinking that you are bewitched and that might just cause enmity between you while the cause is not that*”.

***(Male, 70, Participant 6)***

Adherence to the ESC was impacted by the anticipated stigma associated with performing the standing exercises and daytime elevation (see Table S1).

### 3.8. Sustainability of the ESC

From the perspective of persons affected, challenges related to the sustainability and continued practice of the ESC were attributed to the inability to continue providing medical supplies and halting regular community-based LF-specialist support. This was ultimately limited by internationally funded research coming to an end.

“*You have found me walking around, and then you have received a report that someone with lymphoedema is sick, how would you help him or her after hearing that she or he is sick? Would you send medicine to his or her house or you say she or he will get the drugs when she gets better and return to the hospital?*”

***(Female, 62, Participant 16)***

Additional care and support with practising the ESC was required for those without a caregiver during acute attacks, the elderly, those with additional disabilities (i.e., physical or learning), and for persons with greater disability. More specialist care for moderate-to-severe staging was needed, which was often not available within the routine health system. Additionally, the financial burden of medical supplies for persons affected was greatest for those affected by severe lymphoedema, who needed medical supplies replaced more regularly. Unsustained support following the ESC left many feeling worried, forgotten, and distressed about the future. Additionally, the anticipation of disease progression causing worsening somatic experience, inability to complete daily activities (including the ability to practice ESC), and experienced stigma contributed to continued mental distress following the ESC study.

“*I was thinking that maybe they will no longer supply us again since we were told that they were leaving and maybe they have stopped supplying us with the supplies. So, if they have stopped supplying us, how would they know if our conditions are improving or not?*”

***(Female, 49, Participant 5)***

## 4. Discussion

Syndemics have been rarely discussed in relation to NTDs in comparison to other infectious diseases of poverty such as HIV and TB [1]. The syndemic relationship between NTDs and mental distress has previously been evidenced in Nigeria and Liberia [15,52]. Our study adds to this evidence base, illustrating syndemic interactions between LF and mental distress in Malawi. Within LMICs, the application of syndemic theory should further be explored, as poverty, gender norms, stigma, and weak health systems play an important role within populations’ health and in driving syndemic disease interactions [15]. This study considers the wider biosocial context of LF-related lymphoedema management in Malawi from the perspective of persons affected, which has allowed us to highlight the negative psychosocial consequences of LF and the potential impact of enhanced self-care interventions to address these consequences and contribute to holistic person-centred care. This discussion focuses on interventions at the micro (individual) level, whilst acknowledging there are macro and meso level factors, such as social inequalities related to gender, generation, poverty and extreme climate conditions, which act as a catalyst for the syndemic of concern. We recommend that addressing the social mechanisms driving this syndemic, in addition to enhanced self-care to reverse or prevent physical morbidity associated with the disease, will improve social and health outcomes amongst persons affected by LF.

This study places emphasis on the bidirectional relationship between LF and mental distress. Firstly, we describe the social mechanisms, particularly stigma and stress related to LF, that contribute to mental distress in persons affected. Stress related to LF was exacerbated by stigma, financial insecurities and an inability to work, alongside an absence of appropriate healthcare to diagnose and ‘cure’ their condition. Secondly, we highlight the role of mental distress on the progression of LF symptoms. Depression has been shown to negatively impact health outcomes amongst other chronic diseases, related to health seeking or adherence to treatment [53,54]. Demonstrating this bidirectional relationship between LF and mental distress. Additionally, we found in line with other studies, social determinants such as limited knowledge around the cause of their lymphoedema, and anticipated stigma from healthcare staff were barriers to accessing healthcare [55,56]. Ultimately leading to poor health outcomes for persons affected by lymphedema due to reduced access to diagnosis and treatment [57,58,59,60]. 

To address to the syndemic of LF and mental distress, in Table 2 we give key recommendations for a multi-level system response. Lessons from this work in Malawi can be applied to other endemic countries focusing on holistic care provision for persons affected by LF. In practice, alongside implementing the ESC, we recommend addressing the social mechanisms driving the syndemic. Examples include, establishing peer support groups, stigma reduction interventions and mental health interventions (counselling, peer-counselling, cognitive behavioural therapy, self-help groups and therapeutic workshops) for persons affected and their caregivers, as described in Table 2 [53]. Peer support groups can encourage adherence to self-care, and have been shown to improve wellbeing and self-esteem amongst persons affected by skin-NTDs [61]. Within LF care provision, information and awareness shared with endemic communities and healthcare staff to reduce stigma is crucial in addressing the psychosocial burden of LF. 

To address the social forces that contribute to LF and mental distress clustering, looking beyond the health system, to providing social support through already established social structures is critical. As described in Table 2, providing social support for those living with disabilities, through economic support, socio-economic rehabilitation through skills, educational opportunities and enhancing entrepreneurship, particularly for those that are less physically dependent is recommended [62,63]. Caregiver support to help manage lymphoedema self-care and household responsibili-ties for those with disabilities is recommended. The role of religious groups and traditional healers within social structures and mental health support or care provision is critical in the health-seeking journeys of persons affected by LF and should be considered within future research and intervention design in this area [41,64,65,66]. 

**Table 2:** Overview of Multi-level System Response and Recommendations of LF Programmes for Holistic Care Provision for Persons Affected by LF.

	Recommendations
Early Case Detection	Active case-finding is essential to reduce disease progression, particularly for lymphoedema
Hydrocoele Surgeries	Increased community sensitisation for men to access clinics for hydrocoele surgeries Refresher training for health staff conducting hydrocoele surgery Hydrocoele camps in high endemicity areas
Lymphoedema Management (Self-care)	Scale-up of enhanced self-care activities in districts without trained health staff Periodic refresher training for lymphoedema management for staff, persons affected, and their caregivers Adaption of enhanced self-care activities within the social context of the communities being implemented, i.e., extra provisions for individuals working away on farms to continue practice of self-care Continued provision of supplies (i.e., soap, antifungal cream, painkillers, antibiotics, or ointment, and less frequently towels) Provision of footwear (regularly) Peer-support groups to prioritise and encourage practising of self-care [61] Patient advocates to promote self-care activities [67]
Psychosocial Support	Mental health interventions (counselling, peer counselling, cognitive behavioural therapy, self-help groups, and therapeutic workshops) Stigma reduction interventions, community awareness campaigns [68], stepping stones [69], and community conversations [67] Training clinicians in MhGAP and strengthening mental health referral pathways Provision of rehabilitation services including assistive devices
Social Support	Economic support for persons affected by disabilities Socio-economic rehabilitation through training in skills, educational opportunities, and enhancing entrepreneurship, for persons affected with disabilities, and to contribute towards stigma reduction Caregiver support for those with disabilities and without a carer to manage self-care or household responsibilities Social support given through pre-existing groups or social structures (religious groups, traditional healers, and social groups)
Caregiver Support	Mental health support for caregivers Socio-economic rehabilitation though training in skills, educational opportunities, and enhancing entrepreneurship

These study findings have been member-checked to understand the transferability of this work beyond the Malawian context, which align with WHO policy documents [33] and other researchers within the NTD field working in low-resource settings. For example, DMDI interventions that consider the physical health, mental health and psychosocial care for endemic skin conditions collectively, are currently being piloted in Ethiopia, Nigeria, and Liberia [66,70,71]. Addressing the wider biosocial impact of LF beyond healthcare (i.e., lymphoedema management), has been identified as critical across all these studies. to not just improve physical outcomes of lymphoedema whilst raising awareness of cause, treatment and prevention but also to address the psychosocial consequences of LF, such as reducing stigma and improving mental wellbeing [72]. Within Table S1, we summarise each of the ESC domains (i.e., awareness raising and specific ESC activities) and their intended impact to disrupt the syndemic pathway as well as presenting recommendations for practical actions that can support LF programmes to take a more holistic approach to lymphedema care, including the prioritisation of mental wellbeing alongside physical health.

Access to healthcare services for lymphoedema management and mental health services remains a challenge within Malawi [31]. A lack of trained health staff in lymphoedema management available within health facilities was an additional barrier to accessing appropriate healthcare (i.e., diagnosis and treatment), as evidenced in other LMIC (e.g., India and Sri Lanka) settings [19,73,74,75]. Currently across Malawi, 4000 community health workers and two staff from each health centre (259) have been trained in basic WHO lymphoedema care [29]. Health facility assessments identified that half of the 56 facilities in Malawi had a minimum of one staff member trained in lymphoedema care [29]. More needs to be done in relation to the scale-up of the ESC across all regions as well as regular refresher training of the enhanced self-care. Utilising the community health workforce by upgrading their training to include ESC training should be considered a critical step to improve the availability of accessible, acceptable, and affordable healthcare options for persons affected by lymphedema [75]. Our study has shown that providing ESC support close to communities within highly endemic regions of Malawi [27] can greatly improve mental wellbeing and with appropriate skills building, community health workers may help to address the lack of sustained support in lymphoedema management identified by persons affected in this study. However, such steps should be undertaken with caution so as to adequately support an already highly burdened cadre of the health workforce [76].

This study found that the ESC was able to bring lymphoedema diagnosis and care directly to the community, whilst reducing repeated healthcare-seeking by equipping persons affected with the skills to manage their symptoms [77]. Early case detection for lymphoedema is essential in hindering disease progression to later stages and reducing the severity of disability, functional limitation, and disfigurement [78,79]. Current WHO guidelines are less effective in treating later stages of lymphoedema [36,80,81]. Our findings show that those with more severe staging of lymphoedema experienced increased stigma, mental distress, and disability, as evidenced in previous research [13,14]. Therefore, to meet the needs of persons affected, particularly for those with severe staging of lymphoedema or greater disability, widening the scope of lymphoedema management to include psychosocial support and consider the wider biosocial context within DMDI implementation is critical [82].

The economic context within which DMDI interventions are implemented at the local level is essential [33]. We found key economic barriers to long-term adherence to the ESC, specifically the inability to replace basic supplies for lymphoedema care (e.g., soap, towels, and antifungal cream). Supply replenishment remains a major challenge for many national programmes and is an essential component for providing sustainable DMDI activities [31,77,83,84]. Within settings like Malawi, reducing the economic burden through the provision of medical supplies and suitable footwear to protect feet and prevent infection or wounds is recommended to improve sustained ESC adherence [33]. In addition to this, the promotion of income generation through socio-economic rehabilitation for both persons affected and their caregivers, such as training in skills, educational opportunities, and enhancing entrepreneurship, would also help to address barriers to ESC adherence related to economic instability. Furthermore, lessons from Malawi highlight the importance of supply provision and socio-economic interventions that could be applied to other sub-Saharan endemic countries where persons affected by LF face similar economic climates.

Caregivers should also be recognised as a major contributor to achieving DMDI coverage. Recognising and valuing unpaid care and the domestic work of caregivers and how the absence of this support impacts DMDI coverage is crucial. Significant hardships have been described for the caregivers of persons with lymphedema, most often women [46,85,86]. LF-related lymphoedema has been shown to disproportionately affect women in multiple countries [48,87,88]. For example, in Bangladesh, 3.7 times more women were found to have lymphoedema than men [47]. Such gender inequalities need to be addressed amongst caregivers and persons affected in order to achieve progress towards sustainable development goal 5: “Achieve gender equality and empower all women and girls”. These findings highlight the importance of including the caregiver perspectives within intervention design and LF care delivery.

Qualitative research is labour-intensive and requires rapport-building with participants to collect meaningful and honest accounts. The collection of life history narrative data is dependent upon the skill and patience of the research team as well as the willingness of participants when delivering their accounts. CB was present during data collection as an ‘outsider’ in Malawi; thus, researchers conducted regular debriefs and reflexivity sessions as a team to ensure that honest and transparent interpretations of participant accounts were generated, DEM, as the lead interviewer, established a rapport and trust over time with participants through regular visits during the initial ESC study and in setting up life-history interviews.

## 5. Conclusions

This work illustrates the syndemic interaction of LF and mental distress in Malawi, demonstrating the importance of addressing both the physical and psychosocial consequences of disease within lymphoedema management. Home-based self-care increases the availability of accessible, acceptable, and affordable healthcare options, offering the huge potential to reduce health inequalities whilst enhancing individual agency. This work supports the design and delivery of holistic person-centred DMDI approaches to improve overall health outcomes for people living with LF and/or mental health conditions. Lessons learned can be applied to other sub-Saharan African endemic countries.

## Figures and Tables

**Table 1:** Sampling matrix for life history interviews.

	Mental Distress		Mental Wellbeing Improvement	
	Female	Male	Female	Male
Same or higher acute attacks	2	1	2	2
Reduced acute attacks	3	4	4	2
+1 Male Guardian, trained in the enhanced self-care but was not enrolled onto previous enhanced self-care study.				

## Data Availability

All data generated and analyzed during this study are included in this manuscript. Raw qualitative data are not available and will not be publicly shared, as this would compromise the anonymity and protection of our study participants.

## Supplementary Materials

The following supporting information can be downloaded at: https://www.mdpi.com/article/10.3390/tropicalmed9080172/s1, Table S1: Summary of each enhanced self-care (ESC) domain, the intended impact and outcomes of the intervention from the participant perspective.
