# Supplementary material for: Addressing the Syndemic Relationship between Lymphatic Filariasis and Mental Distress in Malawi: The Potential of Enhanced Self-Care
Source: Trop Med Infect Dis. 2024 Jul 31;9(8):172. doi: 10.3390/tropicalmed9080172 (PMC11360657; doi:10.3390/tropicalmed9080172)
Supplement: Supplementary file 1 [file tropicalmed-09-00172-s001.zip › tropicalmed-3056122-supplementary.pdf]

Table S1: Summary of each enhanced self-care (ESC) domain, the intended impact and outcomes of the intervention from the participant perspective.

Domains of the ESC	Intended Impact on the Syndemic Pathway	Outcome from participant perspective	Recommendations
**Health Awareness**	Clinical rationale to understand the treatment and causes of lymphoedema.	**Reduced internalised stigma.**<br><br>"I was overthinking that maybe it was not good for me to live here on earth because I did not have peace of mind. So, because of that, I was having bad thoughts sometimes because I was feeling that it was not good for me to live since some people were laughing at me." **(Participant 19)**<br><br>"I can say [my mindset has changed] I am used to it and I just consider it as a condition. So, if there are some people that have just met me for the first time … I just take it lightly." **(Participant 2)**<br><br>**Increased awareness of condition and cause, and clinical understanding.**<br><br>*"It is a disease called elephantiasis." **(Participant 16)***	Information and awareness shared with the community to reduce stigma related to ESC activities.<br><br>Community members and health staff provided with health information to reduce enacted stigma.<br><br>Stigma reduction intervention.<br><br>Consideration of religious groups and traditional healers within social structures for care provision.
**Standard WHO activities**			
**Leg washing and drying, attending to lesions/wounds, applying medication**	Prevention of secondary infections, reduce frequency and duration of acute attacks. Maintaining good skin integrity (physical appearance and reduction in odour).	**Reduced acute attacks, reduced fungal infections and healed wounds improving overall appearance of symptoms.**<br><br>*"The other thing is about the cream that you gave us. I have noted that the cream helped me to soften the leg. I was applying it on the stretches that developed on the leg. So, after massaging the leg with the oil, I was also applying the cream and it really helped me out." **(Participant 4)***	Provision of soap – community led or supplied by the health system.

		**Improved daily activities and participation from reduced acute attacks and pain.**<br><br>"I started practising that, things have improved. Right now, I am able to run which was difficult for me in the past … when people told me to move fast, I could just tell them that they should go, I will find them because I was feeling pain." **(Participant 4)**<br><br>**Improved mental wellbeing related to reduced pain, functioning and improved ability to perform daily activities.**<br><br>*"I was stressing out … I was thinking that I cannot do anything … when I had [an] … acute attack, it could take me one week [of]… feeling sick … So, because of that, I could think of just dying because I was feeling like I am a failure. That was then … when I started getting the treatment, I feel like God is answering my prayers."* ***(Participant 5)***<br><br>**Reducing internal and external stigma due to reduced disability.**<br><br>*"They were laughing at me. They were telling me that it was the … beginning of a disability. They have stopped because they have seen me walking."* ***(Participant 4)***	
**Seated exercises**	Mobilisation of ankle, to maintain ankle mobility and improved functioning.	**Easy to practice activity, although many participants were not practicing as had forgotten.**<br>"I have forgotten." **(Participant 15)**	Repeat training to be reminded of activity.

**Day and nighttime elevation**	Reduce swelling and encourage fluid uptake	**Reduction in swelling of the leg, improved physical appearance and functioning.**<br><br>"I should have something for the leg to lean on so that blood can be easily circulating and prevent the swelling of the leg." **(Participant 8).**<br><br>**Reduction in enacted stigma due to reduction in physical disability.**<br><br>"[My family] are happy because in the past, they used to find me here since I was unable even to ride a bicycle … so, when they saw me moving about [after the self-care training] they were happy." **(Participant 4)**	Provision of tools to support adherence of leg elevation (for example, filling an old wheat bag with sand to elevate the leg overnight).<br><br>Horizontal sleeping encouraged and nighttime elevation recommended over daytime elevation.<br>Elevation is most useful in early stages of lymphoedema.
**Managing acute attacks**	Manage acute attacks to reduce pain and inflammatory response.<br>(should cease practice of exercises and massage)	**Improved recovery time from acute attacks resulting in less disruption to daily activities.**<br><br>"The other thing that I was experiencing … my body shakes and the leg could pain me and I was feeling fever as if I have malaria. So, the leg could swell such that I could not walk. Then after 2 weeks, I could get back to normal and the skin of the leg could peel of then I could feel better. So, the process could go on and on. That was it. That was what I was feeling in the past. But since from that time … [we were] taught us the home-based care, I have not been sick … I am able to work as you can see." **(Participant 2)**<br><br>**Reduced repeated health seeking due to ability to manage symptoms.**<br>"After I came back from the hospital and when I noticed that I was not feeling better … I started visiting the	Provision of medication.

		traditional healers. I still visit the hospital … I have tried going to pastors for prayers and for blessed water." **(Participant 20)**	
**Standing exercises**	Build strength in ankle/leg, to help reduce swelling.	**Easy activity to practice.**<br><br>*"Yes, I was told that I should be standing like this and leaning to the wall [demonstrating] and the toes should be partially on the ground"* ***(Participant 6)***<br><br>**Stigma associated with practicing activity.**<br><br>*"I feel that when I start leaning on the wall and do the exercise, people will think that I am going insane"* ***(Participant 2).***	Repeated training and information and awareness shared with the community to reduce stigma related to ESC activities.
**Footwear**	To prevent infection or wounds to feet. Keeping clean and protect from damage.	**Unable to afford shoes.**<br><br>"How can I wear the slippers? I do not have." **(Participant 16)**<br><br>**Additional challenges related to accessibility, e.g. attending the hospital because of lack of footwear.**<br><br>"I do not have slippers and I go there on a foot. There are thorns on the way and I do not see properly, so, everything becomes difficult for me. **(Participant 16)**	Provision of appropriate footwear for persons affected by lymphoedema.
**ESC activities**			
**Deep breathing exercises, lying down exercises (thigh muscle exercises)**	Increased circulation and create pressure so blood and lymph fluid move towards the heart.	**Some found easy to remember and some had forgotten.**<br><br>*"Yes, I have forgotten"* ***(Participant 7).***<br><br>*"For the deep breathing exercise, I still do that"* ***(Participant 5).***	Retraining of deep breathing activity and recommended before bed.

		**Stigma around activities.**	
**Lymphatic massage**	Encouraging lymphatic flow, reduce appearance of swelling.	**Reduced swelling, softened skin and reduced fluid build-up in leg.**<br><br>"The skin peeling stopped …, you taught us to be massaging the leg with the oil and now I have noted that the leg is soft because it was brittle … such that I did not have the hope that my leg would get back to normal again. But when I started massaging it with the oil and the exercises as you told me to be doing, even though I was feeling pain at first but now I am okay. So, the pain is gone and in the coming days, the whole leg will be back to normal." **(Participant 4)**<br><br>**Reducing stigma related to swelling and disability.**	Provision of oil or lotion to promote practicing activity.
**Mobilizing of skin and tissue***	Softening fibrous tissues, improve skin integrity and improving physical appearance.	**Not applicable for participants with mild-stage lymphoedema.**<br><br>**For more severe-stage, disability and illness becomes a barrier to practicing.**<br><br>"When I fall sick [I am unable to practice]" (**Participant 20)**	For participants with more severe lymphoedema, more specialist support required.
**Walking**	To maintain health and mobility and improve functioning and reducing dependency on others.	**Easy for many to incorporate into daily lives.**<br><br>*"They were laughing at me because they were talking a lot about it. They were telling me that it was the beginning of elephantiasis and I will not get better, that was the beginning of a disability. So, that was paining me a lot and I was telling them that the problem was not what they were saying. But then they were insisting that the leg will become bigger … they have*	Recommended walking for all staging of lymphoedema.

		*stopped [talking about me] because they have seen me walking". **(Participant 4)***	
**Eating fresh fruit and vegetables**	Promote wound healing.	**Challenges to provide food.**<br><br>*"Yes, I would want that support .. [with] buying food. Because had it been that I easily find food, I would not have been busy with those other things, I would be busy [looking after] … my leg condition." **(Participant 11)***	Socio-economic rehabilitation though training in skills, educational opportunities and enhancing entrepreneurship.
**Drinking water**	Hydration to improve lymphatic system functioning.	**Not remembered.**	
**Provision of Supplies**			
**Medication cream, soap, oil, hand towels, wash basins**	Ensuring adherence to activities, ability to afford and practice appropriate health care treatment and additional financial burden of supplies to household.	**Providing medical supplies encouraged practicing of ESC activities.**<br><br>"Right now, after the training, I am able to buy drugs if I have money even though at times I struggle when I do not have money." **(Participant 20)** "I face challenges to find soap for washing the leg." **(Participant 5)**<br><br>**Limitations of supply provision impacted adherence once they were finished.**<br><br>*"The soap got finished and with the challenges of finding money, it is difficult to buy the soap sometimes and I fail to take care of the leg because of that" **(Participant 2).***<br><br>*"No, but in the past days I had an acute attack because my drugs got finished"**(Participant 6).***	Continued support with the provision of medical supplies (i.e. soap, antifungal cream, towels, oil).<br><br>Provision of assistive devices to improve physical functioning for those with impaired functioning.<br><br>Provision of one additional basin or water storage container for individuals working away from home (i.e. farming) to encourage continued ESC practice.

* For moderate-severe cases of lymphoedem
